# Two Different Approaches for Oral Administration of Voriconazole Loaded Formulations: Electrospun Fibers *versus* β-Cyclodextrin Complexes

**DOI:** 10.3390/ijms17030282

**Published:** 2016-02-25

**Authors:** Panoraia I. Siafaka, Neslihan Üstündağ Okur, Mariza Mone, Spyridoula Giannakopoulou, Sevda Er, Eleni Pavlidou, Evangelos Karavas, Dimitrios N. Bikiaris

**Affiliations:** 1 Laboratory of Polymer Chemistry and Technology, Department of Chemistry, Aristotle University of Thessaloniki, 54 124 Thessaloniki, Greece; siafpan@gmail.com (P.I.S.); mariza_mone@hotmail.com (M.M.); spiretta@hotmail.com (S.G.); 2 School of Pharmacy, Department of Pharmaceutical Technology, Istanbul Medipol University, Beykoz, 34810 Istanbul, Turkey; nustundag@medipol.edu.tr; 3 School of Pharmacy, Department of Microbiology, Istanbul Medipol University, Beykoz, 34810 Istanbul, Turkey; ser@medipol.edu.tr; 4 Department of Physics, Aristotle University of Thessaloniki, 54 124 Thessaloniki, Greece; etpavlid@auth.gr; 5 Pharmathen S.A, Dervenakion Str. 6, 153 51 Attiki, Greece; ekaravas@pharmathen.gr

**Keywords:** PCL, fibers, inclusion complexation, voriconazole drug, antifungal

## Abstract

In this work, a comparison between two different preparation methods for the improvement of dissolution rate of an antifungal agent is presented. Poly(ε-caprolactone) (PCL) electrospun fibers and β-cyclodextrin (β-CD) complexes, which were produced via an electrospinning process and an inclusion complexation method, respectively, were addressed for the treatment of fungal infections. Voriconazole (VRCZ) drug was selected as a model drug. PCL nanofibers were characterized on the basis of morphology while phase solubility studies for β-CDs complexes were performed. Various concentrations (5, 10, 15 and 20 wt %) of VRCZ were loaded to PCL fibers and β-CD inclusions to study the *in vitro* release profile as well as *in vitro* antifungal activity. The results clearly indicated that all formulations showed an improved VRCZ solubility and can inhibit fungi proliferation.

## 1. Introduction

Recently, it has been estimated that a very large number of population is at risk from fungal infections due to the invasive contamination by *Candida* species [1]. In fact, patients with human immunodeficiency virus (HIV) are the most easily affected group from fungi [2]. A major concern among clinical doctors is to maintain novel strategies so as to prevent antifungal resistance. Antifungal therapeutic agents are mainly categorized to polyene (Amphotericin B), azoles (Fluconazole), and antimetabolites [3]. Amphotericin B was discovered in 1956 and was a gold standard in order to treat fungi infections [4]. Triazoles, a sub-category of azoles are in high importance while have replaced Amphotericin B for the treatment of several fungi species [3]. In most cases triazoles, such as Fluconazole and Ketoconazole, are orally or intravenously administered in order to quickly reach the blood circulation.

Voriconazole [(2*R*,3*S*)-2-(2,4-difluorophenyl)-3-(5-fluoropyrimidin-4-yl)-1-(1*H*-1,2,4-triazol-1-yl)butan-2-ol)] (VRCZ) is a drastic antifungal drug of the azole family with low aqueous solubility (0.71 mg·mL^−1^), which classifies it to BCS class II (Biopharmaceutics Classification System). VRCZ is mainly used for the treatment of several fungi infections, such as invasive aspergillosis, infections from *Candida albicans* and *fusarium* species [5,6]. Its limited solubility in water classified VRCZ as drug with low bioavailability, which limits its effectiveness. This major problem can be solved only by new clinical trends and new pharmaceutical formulations.

Solid dispersions (SDs) are the most easy and interesting techniques for the improvement of drugs solubility and bioavailability enhancement [7,8]. In case of SDs the drug is dispersed in a polymeric matrix, either in amorphous, crystalline or molecularly form. Amongst preparations of the solid dispersions, electrospinning and inclusion complexation using cyclodextrins are the most promising methods [9,10].

Electrospinning is a popular and promising technique for fabrication of nanofibers, due to its simplicity, cost-effectiveness, flexibility, potential to scale up, and ability to spin a broad range of polymers. Electrospinning provides the opportunity for direct encapsulation of drugs into the electrospun fibers with high drug loading [11]. Electrospinning is considered as a simple and versatile process involving an easy apparatus which uses an electrostatic field beneficial to prepare polymer fibers with diameters ranging from nanometers to micrometers [12]. In recent years several biomedical applications include the fibrous structures as scaffolds for tissue engineering and drug delivery systems in view of their fascinating different structures. Analogous with their diameters, porosity, and the used polymer solution different properties arise [13,14]. Aliphatic polyesters like poly(lactic acid), poly(glycolic acid), their copolymers, poly(hydroxy alkanoates) and poly(ε-caprolactone) are the most appropriate candidates for drug delivery systems, due to their low toxicity and biodegradability [15], and can be formulated in fibers via electrospinning technique [16,17,18,19,20].

Additionally, cyclodextrin complexes present significant opportunities as drug delivery systems due to their unique and promising characteristic features [7,9]. Cyclodextrins (CDs) are cyclic oligosaccharides which comprised by glucopyranoside units linked with (1–4) bonds. CDs present a conical shape with an empty cavity which can host small molecules like drugs in proportion with molecules size [21,22,23]. Due to this ability the dissolution behavior of poorly water soluble drugs can be substantially enhanced. Cyclodextrins are used in oral pharmaceutical formulations, by means of inclusion complexes formation, with the following advantages for the drugs: solubility, dissolution rate, stability, and bioavailability enhancement [24].

In the present study, SDs of voriconazole using PCL-encapsulated electrospun fibers and inclusion complex with β-CD were prepared in order to prepare formulations with improved solubility and to compare their release behavior in simulated gastric fluids. In addition, the developed formulations were evaluated for *in vitro* antifungal activity with disc diffusion method. For this reason four different drug loading rates were used (5, 10, 15 and 20 wt %) for each one method.

## 2. Results and Discussion

### 2.1. Morphology of Poly(ε-Caprolactone) (PCL) Fibrous Matrices Loaded and Unloaded with Voriconazole (VRCZ)

**Figure 1:**
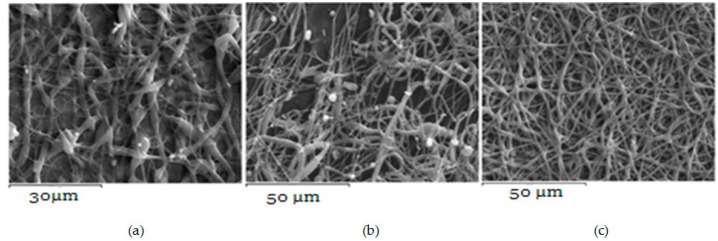
Poly(ε-caprolactone) (PCL) fibers produced by a 20 wt % PCL solution using as solvent: (**a**) DCM; (**b**) DCM/Acetone (70/30 *v*/*v*); and (**c**) DCM/DMF (70/30 *v*/*v*).

PCL fibers were electrospun initially from dichloromethane (DCM), dimethylformamide (DMF), and mixtures of them and further loaded with VRCZ as a model antifungal drug. It was found that the concentration of polymeric solution and the solvent influenced the morphology of the fibers drastically. Simultaneously, major factors during electrospinning process is the supply of voltage and the distance between collector and metallic needle [25,26]. Moreover, the type of solvent has an impact on the morphology of the fibrous matrix because may produce non-uniform fibers. For this reason several solvents and polymer concentrations were tested in our study in order to evaluate the effect of these parameters on fiber preparation. Their surface morphology was characterized by scanning electron microscopy (SEM) in order to understand the way that electrospinning parameters affect the morphology. As can be seen from SEM micrographs presented in Figure 1, the fibrous structures were altered upon the different solvent systems. DCM was found to produce non-uniform large ribbon-like, flat-beaded nanofiber structures (Figure 1a), which is in agreement with the literature [27]. When acetone (Figure 1b) was added to the solution, the structure was also undesirable. On the other hand, the addition of DMF into DCM positively affected the procedure, whereas fibers with average diameter of 729.7 ± 226.2 nm were produced. From preliminary experiments in such solvent mixtures it was found that DCM: DMF (70:30) *v*/*v* (Figure 1c) is the most promised mixture because uniform fibers with narrow average diameter can be produced [28,29].

**Figure 2:**
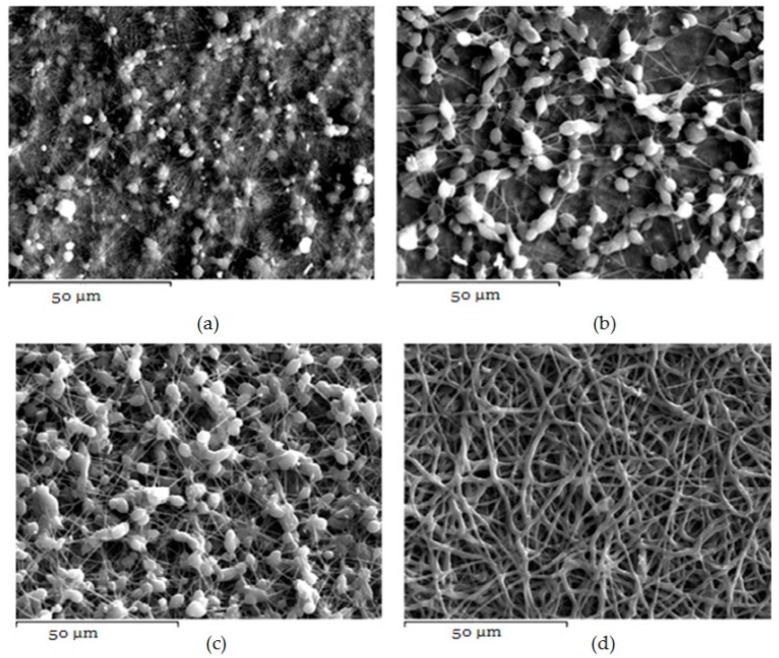
PCL fibers prepared using different concentrations of PCL in DCM/DMF-70/30 *v*/*v* mixture. (**a**) 5%; (**b**) 10%; (**c**) 15%; and (**d**) 20% *w*/*v*.

Additionally, it is largely noted that polymer concentration is critical during the electrospinning process because the procedure is strongly related to the viscosity of the solution. Fabrication and morphology of electospun fibers depend mainly on solution viscosity [30]. Thus, in order to observe any differences on the fibrous matrix related to the concentration, several solutions with different PCL amounts into DCM/DMF mixture (70/30 *v*/*v*) were prepared, which produced fibers with better diameters. Figure 2a–d presents the SEM micrographs and can be seen that diverse structures analogous with the different polymer concentration have been prepared. It can be clearly indicated that in lower concentration (5%–15%) fibers and beads co-existed, and only when the concentration was 20% *w*/*v*, desirable fibrous structures were prepared. The average diameters are 729.7 ± 226.2 nm which is preferable for oral administration. The high surface area of electrospun fibrous matrices enables rapid dissolution and release of its payload in oral delivery systems [31].

**Figure 3:**
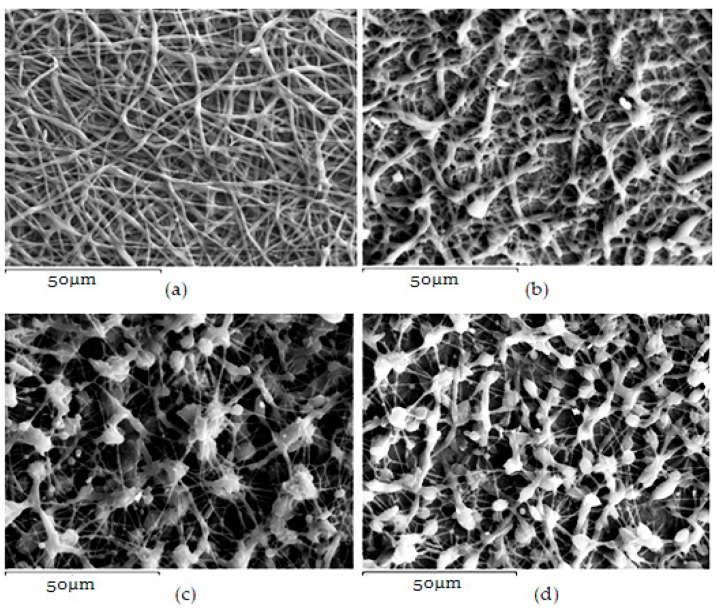
PCL fibers containing: (**a**) 5%; (**b**) 10%; (**c**) 15%; and (**d**) 20 wt % of Voriconazole (VRCZ) drug.

When VRCZ was incorporated in high payloads into the polymer matrix the morphology of prepared fibers was affected dramatically. Only when a low percentage of VRCZ (5 wt %) was added (Figure 3a) the morphology did not changed and is similar to that of neat PCL fibers. Nevertheless, slightly thicker fibers were produced with average diameters 753 ± 237 nm. This fact is in agreement with the current literature [26,32,33]. Researchers have observed that when an additional component such as biomolecule into the polymer/solvent system is entrapped, the solution properties are strongly affected [26,33,34]. As follows increasing VRCZ loading to 10 wt %, a non-uniform and agglomerated architecture was obtained due to the decrease of viscosity, while fiber diameter was further increased. For 15 and 20 wt % loading a beaded structure is seen due to the jet instability. In both concentrations the prepared beads were interconnected with nanofibers.

### 2.2. Morphology, Phase Solubility Studies, and Ultraviolet-Visible (UV-VIS) Spectroscopy of CD Complexes Containing VRCZ

**Figure 4:**
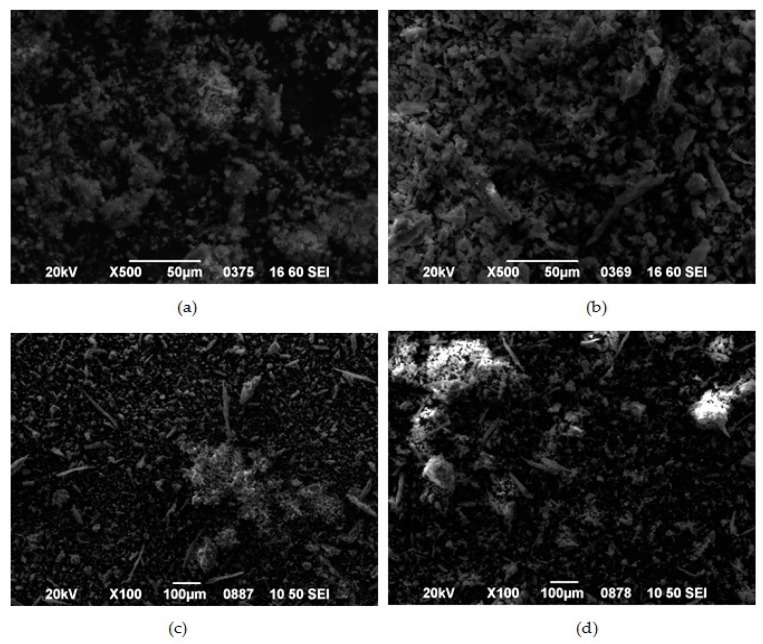
Morphology of β-CD complexes containing: (**a**) 5%; (**b**) 10%; (**c**) 15%; and (**d**) 20 wt % VRCZ.

In the initial stage, for the evaluation of VRCZ L/β-CD complexes SEM analysis was performed (Figure 4a–d) in order to assess the morphology of complexes and their approximate size. From these micrographs, the average size of β-CD + VRCZ particles was measured and is showing at Table 1. As it can be concluded, increment of drug loading resulted to larger particles and it seems that some aggregates were formed after solvent evaporation at freeze drying process. This property plays important role during *in vitro* dissolution. Inducement of the size helps the dissolution to be immediate completed [7]. However, even in such small particle incensement it is expected the drug to be easily released due to the high solubility of β-CD.

Inclusion complexes of β-CDs should be further examined via phase solubility and UV-VIS spectroscopy in order to determine the stoichiometry of the complexes. In addition, absorption spectra from UV-VIS spectroscopy used to confirm the formation of inclusion complex. VRCZ spectra show a peak at 255 nm while β-CD did not present any peak at this area. However, in matter of all the complexes as the drug concentration is increased the intensity of the peak is also increased. Same observations have been reported in the literature confirming the successful inclusion complexation phenomena [35].

**Figure 5:**
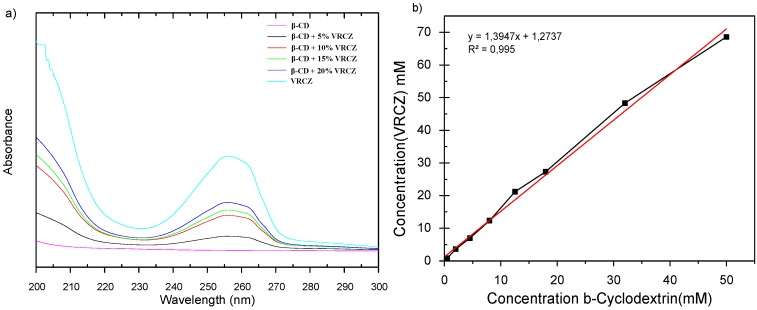
(**a**) UV-VIS spectroscopy and (**b**) phase solubility studies of β-CD/VRCZ inclusion complexes. (Red line is associated as trend line).

**Figure 6:**
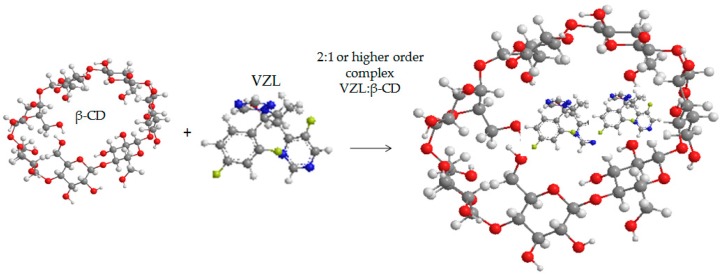
Complexation between VRCZ drug and β-CD.

Phase solubility studies are very critical in order to understand the stoicheometry of the inclusion complexation. From Figure 5b can be observed that as β-CD concentration increased the VRCZ solubility is also increased. Phase solubility curve was found to be of Higuchi’s A_L_ type: that is, a linear increase in drug concentration as a function of increased concentration of β-CD. It has been suggested that when the slope is lower than unity then the formation of 1:1 molecule of CD:drug is involved. However, when slope of the A_L_ isotherm is greater than unity, higher order complexes are assumed to be involved in the solubilization [36]. At these experiments, the slope was calculated at 1.39, confirming the formation of 2:1 or higher complexes (VRCZ /β-CD). The stability constant, *K*c was found at 541 M^−1^ using Equation (1) suggesting that β-CD and VRCZ are having sufficient affinity to form stable inclusion complexes as it is illustrated in Figure 6 [36,37].

### 2.3. Characterization of the VRCZ Formulations Using X-ray Diffraction (XRD) Studies

**Figure 7:**
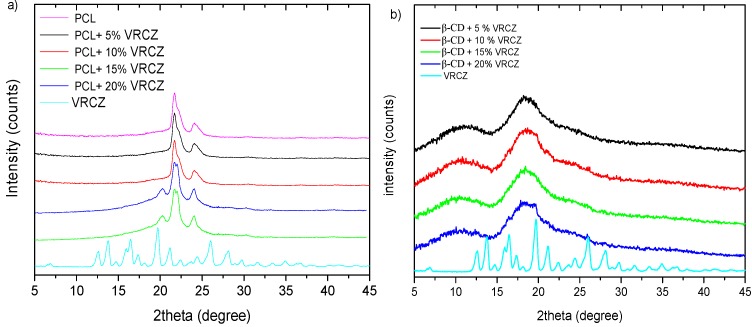
X-ray diffraction patterns of (**a**) PCL fibers containing VRCZ; and (**b**) VRCZ inclusion complexes with β-CD.

In pharmaceutical technology, the formation of amorphous solid dispersion is a crucial factor in order to increase the solubility of the drug [7]. It has been formerly reported that the partial miscibility or poor solubility between drug and polymer can induce the formation of concentrated drug domains which can be recrystallized during the storage period or during the release. XRD measurements were provided for the investigation of VRCZ dispersion into PCL and β-CD matrixes [38]. XRD measurements prove the semi-crystalline nature of PCL due to the presence of two typical peaks at 21.2° and 23.5° (Figure 7) [39]. The crystal structure of PCL electrospun fibers has not been affected by the process, as it was found from other researches [40]. Simultaneously, the diffractogram of VRCZ API consists of sharp multiple peaks at 2θ 12.6°, 13.8°, 16.5°, 17.4°, 19.7°, 21.2°, 24.4°, 26.0°, 28.2°, and 29.9°, indicating the crystalline nature of the drug (Figure 7a) [41]. For the produced PCL/VRCZ loaded fibers the disappearance of the crystalline peaks of VRCZ could be indicated liable decrease in the crystalline nature of the drug inside SD system, which confirms amorphous nature of VRCZ. This amorphization could be attributed to the fine dispersion of VRCZ inside the fibers. Inclusion complexation method could also lead to an amorphous dispersion of the drug onto the CD cavity [7]. In the case of β-CDs/VRCZ (Figure 7b) prepared complexes, XRD patterns reveal that all samples are also amorphous, as in the case of PCL/VRCZ loaded fibers. It is clear that the VRCZ inclusion into β-CD leads to drug amorphization since the corresponded peaks of VRCZ have not been recorded.

### 2.4. Fourier-Transform Infrared (FT-IR) Spectroscopy Studies

**Figure 8:**
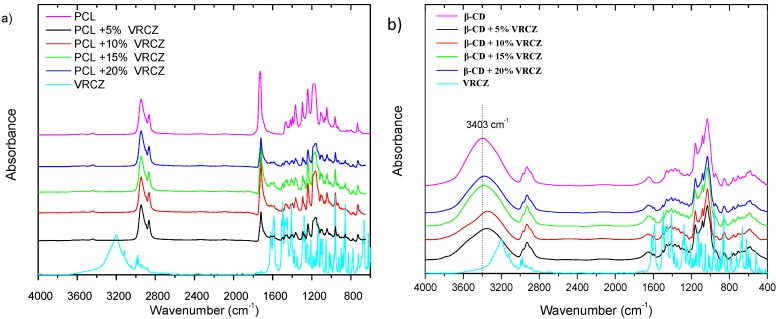
(**a**) FT-IR spectra of PCL fibers containing VRCZ and (**b**) FT-IR spectra of VRCZ inclusion complexes.

In general, drug’s amorphization is resulted due to evolved interactions taking place between the used carriers and the dispersed drug. These interactions lead to API nano-dispersions or molecular level dispersions [7,42,43]. In order to examine possible interactions between VRCZ and PCL as well as with β-CD, FTIR spectroscopy studies were performed. The IR spectra of pure VRCZ showed a broad peak at 3200 and 3121 cm^−1^ due to the hydroxyl groups and aromatic rings, respectively. Peaks at 1615 and 1586 cm^−1^ indicate the aromatic C–C stretch while sharp peaks from 690–515 are associated with the presence of (Fluoro) groups. For PCL fibrous structures, FT-IR spectrum (Figure 8a) of neat PCL showed the typical bands for polyesters at 1756 cm^−1^, which correspond to the carbonyl ester group stretching [39]. In all of the VRCZ-loaded fibers the existence of characteristic peaks of VRCZ drug could suggest its successful loading into fibers. However, shifting or differences on the absorbance of characteristic groups of polyesters and VRCZ L have not been observed to conclude hydrogen bonding or other interactions between carrier and drug. As can be seen from Figure 8a the characteristic peaks of VRCZ and PCL in drug loaded fibers have been recorded exactly at the same positions s in neat materials. For this reason, the amorphization of VRCZ into PCL matrix is attributed to the fine dispersion of it and not to formed interactions.

In contrast to PCL matrices, the β-CD/VRCZ complexes reveal interesting results when compared with the spectra of the pure β-CD and VRCZ drug (Figure 8b). For pure β-CD peaks which observed at 3403 cm^−1^ corresponds to symmetric stretching of hydroxyl groups while the frequency at 2928 cm^−1^ is attributed to the anti-symmetric stretching of CH_2_ band. Inclusion complexes of β-CD and VRCZ could suggest hydrogen bonding between the host and guest molecules. The band at 3403 cm^−1^ as the concentration of the drug is increased is shifted in lower wavenumbers which suggests some interactions [35]. Hence, it is believed that amorphization in β-CD/VRCZ complexes resulted from the extensive hydrogen bonding and the inclusion formation between β-CD and VRCZ, which is in agreement with reported findings in literature [39].

### 2.5. In Vitro Release Studies

In oral delivery, high surface area of electrospun fibrous membrane enables rapid dissolution and release of its payload [44]. Electrospinning provides a very effective tool to increase the surface area and thus speed up the dissolution rate. Since, they possess a highly porous structure that can disintegrate instantaneously when placed in the oral cavity, and release the drugs which then dissolve [45]. Similarly to our study, Nagy *et al.* [46] prepared Donepezil HCl loaded electrospun fibers and they have found that these fibers were showed faster drug release. Additionally, it was mentioned that drugs that have poor water solubility (classes 2 and 4) would improve this property through complexation with hydrophilic CDs or their derivatives and consequently their dissolution rate enhancement [7]. Thus, the inclusion complexes can be used for immediate release applications allowing the drug to dissolve in the GI contents [47].

**Figure 9:**
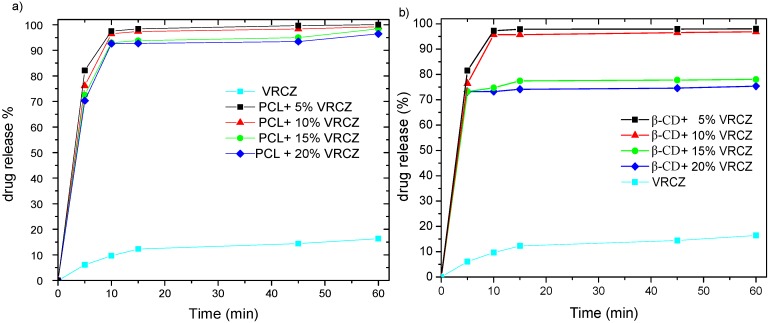
(**a**) *In vitro* release studies of PCL fibers containing VRCZ and (**b**) *in vitro* release studies of VRCZ inclusion complexes.

As it can be revealed from Figure 9 after 1 h only a very small amount of the neat VRCZ drug (10%) was dissolved. This data proves the low solubility of VRCZ in aqueous media and it has been already reported that VRCZ belongs to class II on biopharmaceutics classification system. This problem overcomes incorporating of VRCZ into nanofibers. *In vitro* release studies showed that PCL nanofibers are capable of immediate drug release delivery up to 1 h. In lower drug loading was achieved the higher release rate contribute to the possibility of the drug to dissolved faster. The entire drug was dissolved at 5 min. Some studies evaluate that beads can act as deposits of the drug showing a more sustained release pattern [48]. In the present study, it was shown that the release did not affected by the morphology of the electrospun membranes but probably from the amorphization of the drug [7], or from matrix degradation. As can be seen from Figure 9a the variations between the fibers with different drug loading and drug morphology are slight. In almost all cases an immediate drug release was observed. Table 2 summarizes PCL fibers water uptake and degradation of the fibrous carriers after 1 h of dissolution time. It can be seen that PCL although is a hydrophobic molecule can absorb water at 14% which is attributed to the porosity of the matrix. In addition, the morphology was differentiated after drug release. In case of neat PCL fibrous matrix after 1 h contact with aqueous solution HCl seems to start to disintegrate. For the loaded with VRCZ structures after dissolution some degradation was occurred after 1 h. This could, in cooperation with drug amorphization, to the VRCZ dissolution enhancement.

A similar fast release was also found in β-CD and VRCZ inclusion complexes, especially for 5–10 wt % VRCZ loading (Figure 9b). This could be attributed to the drug amorphization, as was verified by XRD studies. The dissolution profile in these cases is similar to that mentioned for PCL/VRCZ loaded fibers. However, in higher amounts of drug loading a lower dissolution is observed probably due to the formation of bigger particles of the formulation.

### 2.6. Antifungal Activity of Formulations

**Figure 10:**
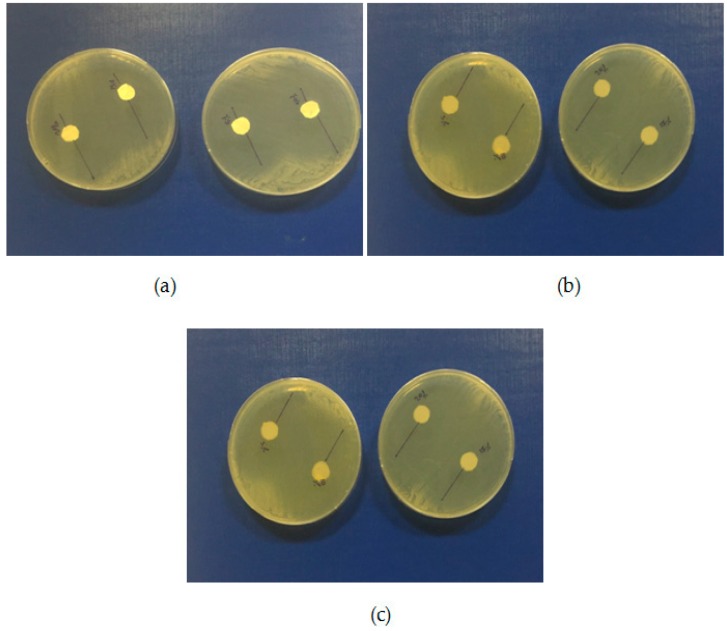
Antifungal activity (**a**) PCL fibers containing VRCZ; (**b**) VRCZ inclusion complexes; and (**c**) VRCZ solution.

The antifungal activities of formulations incorporated with VRCZ were tested against *Candida albicans* ATCC 90028 by disc diffusion method. *Candida albicans* was used, as it is the most common pathogen causing fungal infections. *Candida albicans* is an opportunistic fungal pathogen found as part of the normal microflora in the human digestive tract. The most common body sites showing asymptomatic colonization by *Candida* are the oral cavity, rectum, and vagina [49]. The studies have shown that VRCZ loaded formulations could perform antifungal properties. According to the obtained results (Table 3), VRCZ loaded β-CD complexes and PCL fiber formulation discs were showed similar inhibition zones (Figure 10). Moreover, when the VRCZ concentration was increase the zone diameters were increased depending on concentrations. The β-CD complexes formulations had a slightly higher zone inhibition diameter than PCL fibers, but statistically significant difference has not been determined for antifungal activity between β-CD complexes and PCL fibers against *Candida albicans* (*p* > 0.05). β-CD complex solution was applied to the agar surface with paper; thus, soluble VRCZ has greater ability to diffuse the agar than solid systems. When control and PCL fibers were compared, PCL fibers with insignificant differences were found in zone inhibition diameter. It can be concluded that VRCZ solubility was increased with formulations of fibers and β-CD complexes, soluble drug can show good penetration to agars and similar *in vitro* drug release studies, concerning that in 5–10 min the drug release rate was found approximately 80%. In addition, lower drug loading was achieved the higher release rate due to the possibility of the drug to be dissolved faster. This is in agreement with dissolution studies mentioned previously. In this study, lower drug loadings shows higher release and penetration, as well as good antifungal activity. For example, 5% drug loading present 100% release rate in first minutes in addition with the zone inhibition zones was found 4.1 cm for β-CD and 4.0 cm for PCL fibers. Accordingly, similar results were found for all formulations.

## 3. Experimental Section

### 3.1. Materials

PCL with *M*_W_ = 80.000 g/mol and the solvents dichloromethane (DCM), *N*’-*N*-dimethyl formamide (DMF) and acetone were purchased from Sigma-Aldrich (Steinheim, Germany). Voriconazole drug was kindly donated from Pharmathen S.A. (Athens, Greece). All other reagents were of analytical grade.

### 3.2. Fabrication of the Electrospun Fibers

PCL fibers were prepared via an electrospinning technique. PCL was dissolved in DCM, DMF, and mixtures of these solvents preparing solutions with different polymer concentrations ranged from 5 to 20 wt %, in order to achieve the desirable fiber morphology. For the preparation of drug loaded fibers PCL and VRCZ at 5, 10, 15, and 20 wt % drug loading were added to a DCM/DMF 70/30 *v*/*v* solvent mixture preparing 20% *w*/*v* homogeneous solutions with constant stirring. The solutions were injected from electrospinning device using a metallic rotating cylindrical drum as a collector. Voltage was kept at 16 kV, the distance between needle and collector was 7 cm, and the flow rate was 1.5 mL/h.

### 3.3. Preparation of Inclusion Complex of VRCZ into β-CD via Freeze-Drying Method

VRCZ and β-CD were dissolved separately in a mixture of acetonitrile and distilled water. The solutions were mixed with different molar ratios in order to prepare formulations with 5, 10, 15, and 20 wt % drug loading. The solutions were magnetically stirred for 24 h at room temperature and after that were freeze dried. After solvent removal the obtained white colored material was pulverized in order to obtain a powder.

### 3.4. Phase Solubility Studies for Inclusion Complexation

Phase solubility studies were carried out in water in triplicate according to the method described by Higuchi and Connors [50]. Excess amount of VRCZ (50 mg) was added to 20 mL of aqueous solutions containing various concentrations of β-CD (0–0.01 M). Then, the suspensions were shaken on a rotary shaker at 25 ± 2 °C for four days. After equilibrium was achieved, the samples were filtered through 0.45 m membrane filter and appropriately diluted. The concentration of VRCZ was determined spectrophotometrically (Shimadzu UV-VIS Spectrophotometer, 1700, Tokyo, Japan) at 255 nm. The apparent stability constants *K*s were calculated from phase solubility diagrams with the assumption of 2:1 stoichiometry according to the following equation:

(1) $$slope\;=\;\frac{2S_{0}K_{2:1}}{S_{0}^{2}K_{2:1}}$$

where *S*_o_ is the solubility of VRCZ in absence of β-CD.

### 3.5. Characterization of Formulations

The prepared electrospun fibers and β-CD complexes loaded with VRCZ were characterized using different techniques, such as scanning electron microscopy (SEM, model Zeiss Supra 55 VP, Jena, Germany), infrared spectroscopy (FTIR–2000, Perkin Elmer, Dresden, Germany) and X-ray diffraction (XRD, model Rigaku-Miniiflex II, Tokyo, Japan) in order to identify the morphology of the formulations. *In vitro* release studies of the drug were performed in order to evaluate the enhancement of VRCZ.

#### 3.5.1. Surface Morphology

Surface morphology of the prepared formulations was evaluated using a scanning electron microscope (SEM) (model Zeiss Supra 55 VP, Jena, Germany). The accelerating voltage was 15.00 kV and the scanning was performed *in situ* on a sample powder. All the data were analyzed via Digimizer software in order to assess the mean diameter of the fibers and particle sizes of VRCZ/β-CD complexes.

#### 3.5.2. X-ray Diffraction Studies

X-ray powder diffraction (XRD) patterns were recorded a XRD–diffractometer (Rigaku-Miniiflex II, Tokyo, Japan) with a CuKα radiation for crystalline phase identification (λ = 0.15405 nm for CuKa). The sample was scanned from 5° to 45°.

#### 3.5.3. FT-IR Spectroscopy

FTIR spectra of the samples were taken with a FTIR-spectrometer (model FTIR–2000, Perkin Elmer, Dresden, Germany) using KBr disks (thickness of 500 μm). The spectra were recorded from 4000 to 400 cm^−1^ at a resolution of 2 cm^−1^ (64 co-added scans). The spectra presented are baseline corrected and converted to the absorbance mode.

#### 3.5.4. Evaluation of Drug Entrapment and Drug Loading

Drug loading efficiency in the formulations was assessed by UV-VIS spectrometer (model LC-20AD). The concentration of drug in the solution was obtained from the standard curve of VRCZ in acetonitrile (ACN):H_2_O, which relates absorbance and concentrations. Briefly, 10 mg of the samples were dissolved in 10 mL ACN:H_2_O and were extracted on shaker for 24 h at 100 rpm. The solutions were then filtrated and VRCZ content in was determined by UV-VIS spectrometer. The loading efficiency of drug in formulations was calculated using the equations:

(2) $$\text{Drug loading content (\%)}\;=\;\frac{weight\;of\;drug\;in\;SDs}{weight\;of\;SDs}\;\times 100$$

#### 3.5.5. *In Vitro* Drug Release

For the *in vitro* release studies, a dissolution apparatus type DISTEK E-3400 evolution system, equipped with an autosampler using the paddle method (USP II method) was used. Each dissolution vessel was loaded with quantity of formulations corresponding to 100 mg of VRCZ. The test was performed at 37 ± 1 °C with a rotation speed of 50 rpm. The dissolution medium was 500 mL of HCl 1 N.

#### 3.5.6. Disk Diffusion Testing

Antifungal activity of formulations was determined by a disc diffusion method according to the guidelines of the Clinical and Laboratory Standards Institute (CLSI). For disc diffusion testing, 90-mm-diameter plates containing Mueller Hinton Agar at a depth of 4.0 mm were used. *Candida albicans* ATCC 90028 was incubated (NUVE EN120, Istanbul, Turkey) on Sabouraud’s dextrose agar for 24 h. After the incubation, microbial suspension was prepared in 5 mL of sterile 0.85% saline. The resulting suspension was VRCZ vortexed and the turbidity of the suspension was adjusted to yield 1 × 10^6^–5 × 10^6^ cells/mL (0.5 McFarland standard). The agar surface was inoculated by using a swab dipped in a cell suspension adjusted to the turbidity of a 0.5 McFarland standard. The inoculum was allowed to dry for 15 min. To apply onto the plates VRCZ (5%, 10%, 15%, and 20%) loaded PCL fibers has been cut (0.6 cm diameter) and each the 0.6 cm diameter discs were carefully placed onto the inoculated plate surfaces. VRCZ (5%, 10%, 15%, and 20%)-loaded β-CD complexes were weighted and dissolved. The β-CD complexes solution was dropped to the sterile paper discs (Wattman filter no. 42, diameter of 6 mm) and paper discs were carefully placed onto the surfaces of the inoculated plates at suitable distance with the help of sterile pointed forceps. In addition, VRCZ solution was used as a positive control. VRCZ solutions were prepared and dilutions were made to adjust the drug amount. The VRCZ solution was dropped to the sterile paper discs and paper discs were carefully placed onto the surfaces of the inoculated plates. Two discs were placed in each Petri dish. Then, the plates were incubated in air at 37 °C for 48 h. Each test was performed in triplicate and the mean zone size was determined [51,52].

## 4. Conclusions

To evaluate and compare the efficiency of two different drug delivery systems for the antifungal drug known as VRCZ, solid dispersions based on PCL electrospun nanofibers and β-CD complexes were successfully developed by using an electrospinning technique and lyophilization method, respectively. Scanning electron micrographs revealed that the nanofibrous morphology affected by various parameters such as solvent kind and polymeric concentration. Homogeneous cylindrical fibers are prepared using DCM/DMF mixture (70/30 *v*/*v*) and 20 wt % PCL concentration. However, at 10–20 wt % of VRCZ loading a beaded structure is seen. Drug loading was high in all cases due to the large surface area for nanofibers. Regards to β-CD complexes, fine and amorphous dispersion of VRCZ was achieved, as was revealed by XRD studies due to hydrogen bonding between CD and VRCZ L. *In vitro* release studies showed in both formulations an immediate release, less than 60 min, due to drug amorphization. Antifungal activity was examined in common species of *Candida albicans* and showed high efficiency in regard to the VRCZ addition. Comparing the release rate and antifungal properties of the prepared formulations it can be seen that both are effective with very small differences reveling that both can be used as appropriate carriers for VRCZ drug.

## Figures and Tables

**Table 1:** Particle sizes of β-CD complexes loading with Voriconazole (VRCZ).

Sample	Particle Size (μm)
β-CD + 5 wt % VRCZ	2.14 ± 0.75
β-CD + 10 wt % VRCZ	3.42 ± 1.56
β-CD + 15 wt % VRCZ	12.02 ± 4.9
β-CD + 20 wt % VRCZ	25.26 ± 8.8

**Table 2:** Water uptake and degraded morphology of the PCL/VRCZ fibers during dissolution for 1h.

Sample	Water Uptake (%)	Morphology after Dissolution (HCl 1N)
PCL	14.578 ± 0.903	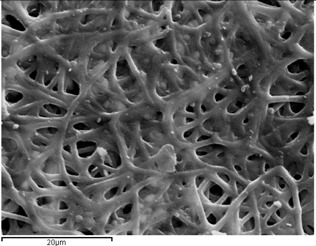
PCL + 5% VRCZ	15.789 ± 0.688	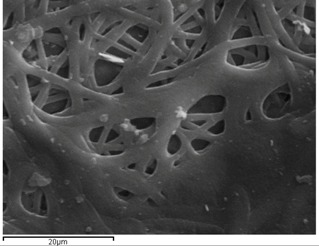
PCL + 10% VRCZ	15.2723 ± 1.114	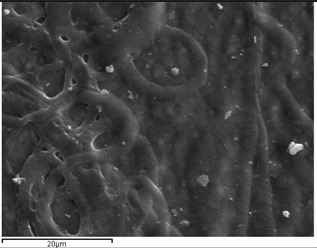
PCL + 15% VRCZ	15. 645 ± 1.235	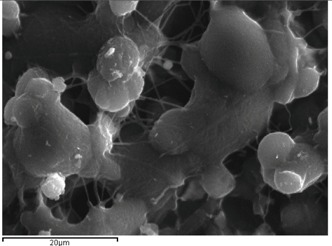
PCL + 20% VRCZ	14.306 ± 1.908	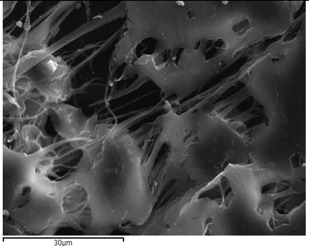

**Table 3:** Results of the formulation zone inhibition.

VRCZ Concentration	Zone Inhibition Diameter (cm)		
	Control	β-CD Complexes	PCL Fibers
5%	4.0 ± 0.3	4.1 ± 0.2	4.0 ± 0.3
10%	4.6 ± 0.1	5.2 ± 0.1	4.8 ± 0.2
15%	4.8 ± 0.2	5.4 ± 0.2	5.2 ± 0.3
20%	5.0 ± 0.2	5.4 ± 0.3	5.4 ± 0.1
